# LncRNA CASC2 Regulate Cell Proliferation and Invasion by Targeting miR-155/SOCS1 Axis in Hepatocellular Carcinoma

**DOI:** 10.1155/2023/8457112

**Published:** 2023-02-10

**Authors:** Ye Yuan, Jiaxin Ye, Xujing Zhang, Zhisu Liu

**Affiliations:** ^1^Department of Hepatic and Gall Surgery, Zhongnan Hospital of Wuhan University, Wuchang, Wuhan 430000, China; ^2^Health Examination Center, Wuhan Third Hospital, Tongren Hospital of Wuhan University, Wuchang, Wuhan 430000, China

## Abstract

Long noncoding RNAs (lncRNAs) have been reported to be involved in the development and progression of various human malignancies. However, the role of lncRNA CASC2 in hepatocellular carcinoma (HCC) remains mostly unknown. The aim of this study was to investigate the potential roles and underlying mechanisms of CASC2 in HCC progression. We found that CASC2 expressions were downregulated in HCC tissue samples and cell lines. The clinical assays revealed that lower levels of CASC2 were associated with the TNM stage, lymph node metastasis, and a poorer prognosis specific to HCC patients. Overexpression of CASC2 inhibited the proliferating, migratory, and invasion capacity of HCC cells. Bioinformatics analysis and the luciferase reporter assay revealed that CASC2 worked as a molecular sponge for miR-155. And CASC2 could upregulate SOCS1 expression by inhibiting miR-155 expression in HCC cells. Furthermore, SOCS1 inhibition partially inverses the suppression effect of cell proliferation, migration, and invasion regulated by CASC2 in Huh7 and HepG2 cells. Taken together, our findings identified CASC2 as a tumor suppressor to inhibit HCC development by regulating the miR-155/SOCS1 axis, and CASC2 might be a potential therapeutic target of HCC for future clinical treatment.

## 1. Introduction

Hepatocellular carcinoma (HCC) is one of the most prevalent malignant tumors with a high morbidity and mortality rate worldwide. Due to the lack of diagnostic biomarkers and other methods for detecting HCC in the early stages, many HCC patients are diagnosed at an advanced stage, and some effective interventions like surgery and liver transplantation are not suitable to conduct [1]. Although there are more and more advances in therapy for treating HCC, such as surgical operation, radiotherapy, and molecularly targeted therapy, the 5-year survival rate of HCC patients still remains dismal [2]. Tumor metastasis and recurrence after surgery are the main reasons which lead to the poor prognosis [3]. Hence, it is urgent to investigate the underlying molecular mechanisms of HCC and explore novel therapeutic targets to improve the diagnosis and prognosis of HCC patients.

Long noncoding RNAs (lncRNAs) are a class of noncoding RNAs longer than 200 nucleotides and have no protein-coding capacity. In recent years, accumulating studies have shown lncRNAs play an essential role in a broad range of cellular mechanisms like transcriptional activation, intranuclear trafficking, and epigenetic modifications [4]. It has been reported that lncRNAs are differently expressed in many cancers and play essential roles in tumorigenesis by regulating oncogenes or tumor suppressor genes, which prompts growing interest in the potential clinical application as therapeutic targets of tumors. Some well-studied lncRNAs such as HOX Transcript Antisense RNA (HOTAIR), X-Inactive Specific Transcript (XIST), and Nuclear Enriched Abundant Transcript1 (NEAT1) [5–7] demonstrated to participate in tumor progression, including regulating cell proliferation, cell cycle, apoptosis, and metastasis.

LncRNA Cancer Susceptibility Candidate 2 (CASC2) was first found in endometrial cancer in 2004 and could inhibit endometrial cancer carcinogenesis, which serves as a tumor suppressor [8]. Recent studies indicate that CASC2 is expressed at low in many human malignancies including cervical cancer, pancreatic cancer, and thyroid cancer [9–11]. In gastric cancer, CASC2 acts as a tumor suppressor, and overexpression of CASC2 could inhibit proliferation, colony formation, migration, and invasion of cancer cells [12]. Moreover, CASC2 was found downregulated in malignant melanoma, and low CASC2 expression was correlated with tumor size, TNM stage, and poor overall and disease-free survival (DFS) of malignant melanoma patients [13]. CASC2 also acts as a ceRNA for miR-18a-regulated STAT3 expression and inhibits the proliferative and metastatic capability of colorectal cancer cells [14]. And it is reported that CASC2 may affect cell growth and apoptosis of HCC cells through different mechanisms [15]. However, the involvement of CASC2 in the regulation of HCC pathogenesis remains largely unknown.

In this study, we confirmed the low expression of CASC2 in HCC tissues and cell lines and explored the regulatory effect of CASC2 on cell proliferation, migration, and invasion. Furthermore, we revealed that CASC2 could serve as the miR-155 sponge and promote the expression of suppressor of cytokine signaling 1 (SOCS1). Our findings suggest that CASC2 might inhibit the progression of HCC by regulating the miR-155/SOCS1 axis.

## 2. Materials and Methods

### 2.1. Clinical Specimens

42 pairs of HCC tissues and adjacent normal tissues were obtained from Zhongnan Hospital of Wuhan University between May 2018 and October 2019. All samples were immediately frozen in liquid nitrogen after surgery and stored at −80°C. The specimens were confirmed by two senior independent pathologists, and tumor staging was performed following the tumor node metastasis (TNM) staging method of UICC and AJCC 2008. Subjects were excluded if they exhibited any evidence of other malignancies or any other type of liver disease. And all HCC patients were free from human immunodeficiency virus (HIV), hepatitis virus and any other viral infections. All patients did not receive radiotherapy, chemotherapy, or any other antitumor therapy before surgery. This study was approved by the ethics committee of Zhongnan Hospital of the Wuhan University. Written informed consents were obtained from all participants before the study.

### 2.2. Cell Culture

Human HCC cell lines (HepG2, Huh7, SMMC-7721, and QGY-7701) and the normal liver cell line LO2 were obtained from the American Type Culture Collection (ATCC, Manassas, VA, USA). The cells were cultured using Roswell Park Memorial Institute 1640 (RPMI 1640) medium (Gibco, Rockville, MD, USA) supplemented with 10% fetal bovine serum (Gibco, Rockville, MD, USA), 100 U/ml penicillin, and 100 U/ml streptomycin. All cells were in a humidified incubator at 37°C and 5% CO_2_.

### 2.3. Cell Transfection

MiR-155 mimics and miR-con were obtained from RiboBio (Guangzhou, China), and 100 pmol miR-155 mimics were transfected into each well, respectively. Plasmids with the pcDNA3.1 vector (Invitrogen, Carlsbad, CA, USA) containing CASC2 and SOCS1 overexpression sequences were constructed, and 2 *μ*g plasmid were transfected into each well. Transfection of HCC cells was conducted using Lipofectamine 3000 (Thermo Fisher Scientific, Waltham, MA, USA) following the manufacturer's instructions. Cells were harvested after 48 h transfection for subsequent experiments.

### 2.4. qRT-PCR Assay

Total RNA was extracted from HCC tissues, adjacent tissues, and HCC cells using TRIzol reagent (Invitrogen, Carlsbad, CA, USA). Then the reverse transcription kit (Invitrogen, Carlsbad, CA, USA) was used to reverse-transcribe the RNA into cDNA. The QRT-PCR assay was performed using the SYBR Green Real-Time Kit (TaKaRa, Tokyo, Japan) and real-time PCR system (Applied Biosystems 7500, Foster City, CA, USA) according to the manufacturer's guidelines. U6 was used to normalize the relative expression of CASC2 and miR-155, and GAPDH was used to normalize the relative expression of SOCS1. The relative fold changes of target genes were evaluated by the 2^−∆∆Ct^ method. lncRNA CASC2 forward, 5′-GCT CGG ACG AAG ATT GGA GA-3′ and reverse, 5′-ATA AGG TCA GTA ATG AGA ACT GC-3'; U6 forward, 5′-CTC CTT GTA AGC ATT GAG T-3′ and reverse, 5′-AAC AGG CAG TTT ACG CGC TC-3'; GAPDH forward, 5′-AGT GTC ACC GTT CAG CCC TTG-3′ and reverse, 5′-ACC AAG TTG CAA CAG GTC AAG-3′.

### 2.5. CCK-8 Assay

The Cell Counting Kit 8 (CCK-8) assay (Solarbio, Beijing, China) was used to detect cell proliferation ability. Approximately 1 × 10^4^ Huh7 cells were seeded in each well of 96-well plates overnight at 37°C. 10 *μ*l of CCK-8 reagent was added to each well after incubation. After incubating for another 2 h at 37°C, the absorbance at 450 nm was determined by a microplate reader (Enspire, USA), and all results were recorded.

### 2.6. Transwell Assay

Transwell assays were used to evaluate the cell migration and invasion abilities. For the migration assay, 1 × 10^5^ cells were suspended in 500 *μ*l serum-free RPIM-1640 medium and seeded into the upper chamber (Corning, Corning, NY, USA), and 700 *μ*l RPIM-1640 containing 10% Fetal Bovine Serum(FBS) was added to the lower chamber. After 24 h incubation at 37°C, the cells on the lower surface were fixed with paraformaldehyde, stained with 0.2% crystal violet, and then imaged and enumerated with an inverted microscope (Nikon, Tokyo, Japan). For invasion assay, the upper chamber was coated with Matrigel (BD, Franklin Lakes, NJ, USA) before cells seeded, and the other steps were the same as for migration assay.

### 2.7. Luciferase Reporter Assay

The potential complementary sequences of CASC2 and miR-155 were predicted using StarBase (https://starbase.cysu.edu.cn/). Sequences of wild-type (Wt) or mutant-type (Mut) CASC2 or 3′UTR of SOCS1 were synthesized and cloned into the commercial pmirGLO reporter vectors (ThermoFisher, Waltham, MA, USA). The vectors were cotransfected with miR-155 mimics or miR-NC into 239T cells with Lipofectamine 2000. Subsequently, the luciferase assays were performed using the dual-luciferase reporter assay system (Promega, Madison, WI, USA) after 24 h of transfection, according to the manufacture's protocols.

### 2.8. Western Blot Analysis

Radioimmunoprecipitation assay (RIPA) lysis buffer (Beyotime, Shanghai, China) was used to extract the total proteins from HCC tissues and cell lines, and the protein concentrations were quantified by bicinchoninic acid (BCA) assay (Pierce, Rockford, IL, USA). 50 *μ*g of total proteins were separated using 10% sodium dodecyl sulfate-polyacrylamide gel electrophoresis (SDS-PAGE) and transferred to a polyvinylidene fluoride (PVDF) membrane (Millipore, Billerica, MA, USA). After incubating with nonfat milk for 2 h at room temperature to block the membrane, the PVDF membrane was incubated with primary antibody (Abcam, Cambridge, MA, USA) at 4°C overnight, and then they were incubated with secondary antibody. Finally, proteins were visualized using diaminobenzidine (DAB) chromogenic kit (Solarbio, Beijing, China), and the intensity of bands was quantified using Image J software (NIH, Bethesda, MD, USA).

### 2.9. Statistical Analysis

All data were analyzed using the statistical product and service solutions (SPSS) 22.0 software (IBM, Armonk, NY, USA) and presented as the mean ± standard deviation $\left(\bar{x}\pm s\right)$. The Student's *t*-test was used to analyze the significance of the difference between two groups. The Spearman's correlation analysis was used to analyze correlation among genes. *P* < 0.05 was considered as statistically significant difference.

## 3. Results

### 3.1. CASC2 is Highly Expressed and Affect the Clinical Progress of HCC

**Figure 1:**
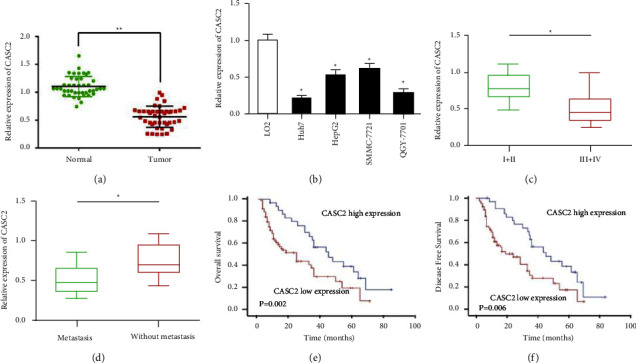
CASC2 was downregulated in HCC and correlated with clinical parameters. (a) The expression of CASC2 in HCC samples. (b) Relative CASC2 expression in four HCC cell lines and normal liver cell line. (c) Relative CASC2 expression in HCC tissues with different TNM stages. (d) Relative CASC2 expression in HCC tissues with or without metastasis. (e)-(f) Kaplan-Meier assays for OS and DFS curves according to CASC2 expression levels. ^*∗*^*P*  <  0.05, ^*∗∗*^*P*  <  0.01.

Quantitative real-time PCR was used to identify the expression of CASC2 in HCC tissues and cells. The results showed that CACS2 was significantly decreased in HCC tissues compared to adjacent normal tissues (Figure 1(a)). Similarly, we also found the expression of CASC2 was distinctly low-regulated in HCC cell lines (HepG2, Huh7, SMMC-7721, QGY-7701) compared to normal liver cell LO2 (Figure 1(b)). Then the 42 patients were divided into 2 groups depending on the median value of CASC2 expression (low CASC2 group *n* = 21; high-CASC2 group *n* = 21). As the results showed, low expression of CASC2 presented a positive correlation with an advanced TNM stage (Figure 1(c)) and positive lymph node metastasis (Figure 1(d)). To explore whether CASC2 may influence the survivals of HCC patients, Kaplan–Meier assays were performed, and the results showed that patients with high-CASC2 expression possessed longer overall survival (OS) and disease-free survival (DFS) compared to the low-CASC2 group (*P* < 0.05). Furthermore, univariate analyses showed that CASC2 expression, TNM stage, and lymph node metastasis were significantly associated with OS and DFS. Moreover, multivariate analyses revealed that CASC2 expression was an independent prognostic indicator of HCC patients regarding OS and DFS (Table 1), suggesting CASC2 might be a promising biomarker of prognosis.

### 3.2. Overexpression of CASC2 Inhibits HCC Cell Proliferation, Migration, and Invasion in HCC Cells

**Figure 2:**
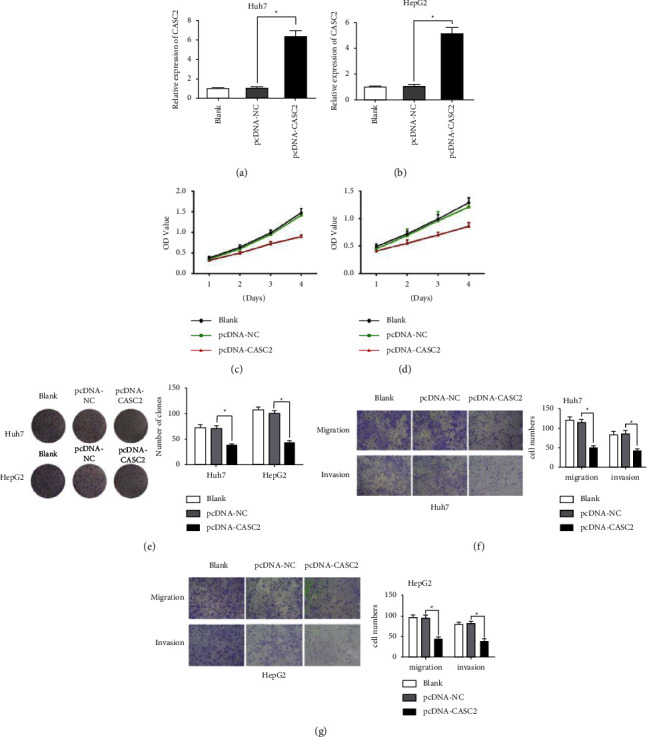
Biological functions of CASC2 on HCC cell proliferation, migration and invasion. (a) CASC2 expression in Huh7 and HepG2 cells were measured after transfected with pcDNA-NC or pcDNA-CASC2. (c)-(d) Growth curve of Huh7 and HepG2 cells by CCK-8 assays. (e) Colony formation of Huh7 and HepG2 cells. (f)-(g) The changes of migration and invasion ability in Huh7 and HepG2 cells after transfection. ^*∗*^*P*  <  0.05, ^*∗∗*^*P*  <  0.01.

To validate the biological role of CASC2 in HCC cells, the Huh7 and HepG2 cell lines were chosen for functional experiments due to their low CASC2 expression. As shown in Figure 2(a), the expression of CASC2 in Huh7 and HepG2 cell lines were significantly upregulated after cell transfection. The CCK-8 assay demonstrated that overexpression of CASC2 significantly suppressed the proliferative abilities of Huh7 and HepG2 cells (Figure 2(b)). Furthermore, the colony formation assay showed that overexpression of CACS2 significantly decreased the number and size of cell colonies (Figure 2(c)). Transwell migration and invasion assays revealed that after CASC2 was overexpressed, the number of cells penetrating the inserts was significantly decreased, indicating that CASC2 could obviously inhibit the migratory and invasive capabilities of Huh7 and HepG2 cells (Figures 2(d) and 2(e)).

### 3.3. CASC2 Is a ceRNA and Functions as a Molecular Sponge for miR-155

**Figure 3:**
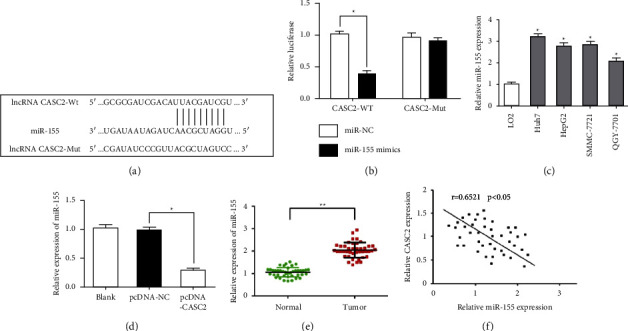
(a) The binding sites between CASC2 and miR-155 were predicted by StarBase 2.0. (b) Dual-luciferase reporter assays were conducted to confirm that CASC2 could target miR-155 directly. (c) The expression of miR-155 was detected by RT-qPCR in HCC cells. (d) Overexpression of CACS2 suppressed the expression of miR-155. (e) The expression of miR-155 was detected by RT-PCR assays. (f) The correlation analysis between CASC2 and miR-155 expression in HCC samples. ^*∗*^*P*  <  0.05, ^*∗∗*^*P*  <  0.01.

To reveal the underlying molecular mechanism of CASC2 in regulating HCC cells, the bioinformatics analysis website StarBase 2.0 was adopted to predict the downstream target of CASC2. The results showed there was a binding sequence between CASC2 and miR-155 (Figure 3(a)). Dual-luciferase reporter assay indicated that miR-155 mimics could weaken the luciferase activities when transfected with the Wt-CASC2. However, miR-155 mimics did not reduce the luciferase activities when transfected with the Mut-CASC2, which indicated that CASC2 could bind to miR-155 directly (Figure 3(b)). In addition, we observed the distinct increase in miR-155 expression in 4 HCC cell lines (Figure 3(c)). We also found that overexpression of CASC2 inhibit the level of miR-155 in Huh7 cells markedly (Figure 3(d)). Moreover, miR-155 was upregulated in HCC tissues compared to adjacent normal tissues (Figure 3(e)). Pearson's correlation analysis was used to analysis the relationship between the expression of CASC2 and miR-155 in HCC specimens. The results showed the expression of CASC2 was negative correlated with miR-155 (Figure 3(f)). Taken together, our findings indicated that CASC2 acted as a competing endogenous RNA to sponge miR-155 in HCC cells.

### 3.4. SOCS1 Acts as a Target Gene regarding miR-155

**Figure 4:**
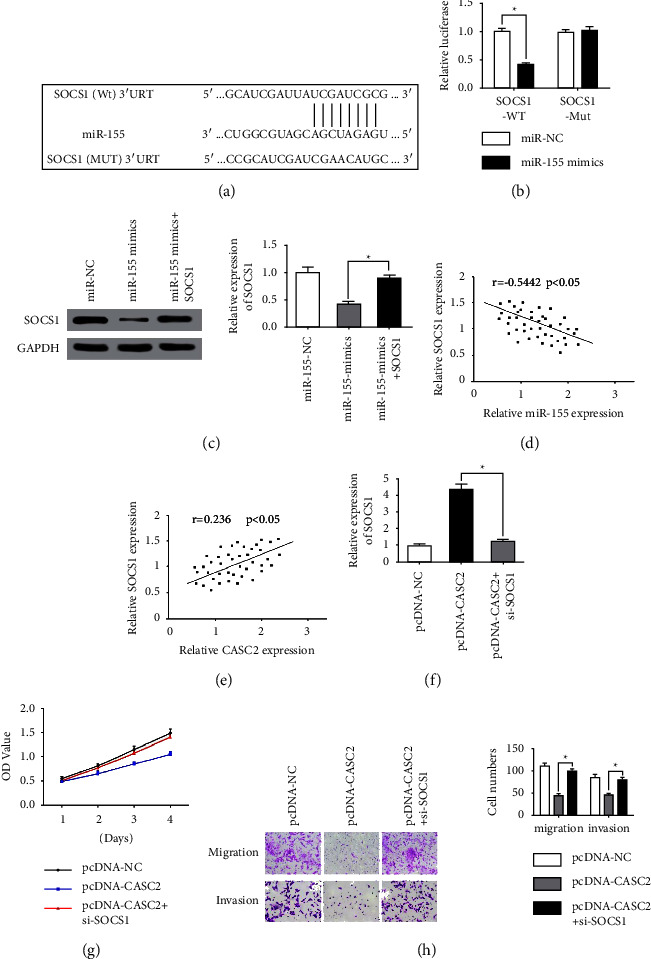
(a) The binding sites between SOCS1 and miR-155 were predicted by StarBase 2.0. (b) Dual-luciferase reporter assays were conducted to confirm that Mir-155 could target SOCS1 directly. (c) The mRNA and protein expressions of SOCS1 after transfection. (d) The correlation analysis between SOCS1 and miR-155 expression in HCC samples. (e) The correlation analysis between SOCS1 and CASC2 expression in HCC samples. (f) Protein expression changes of SOCS1 after transfection. (g) Growth curve of Huh7 cells by CCK-8 assays after co-transfection. (h) The determination of the effects of SOCS1 on the migration and invasion. ^*∗*^*P*  <  0.05, ^*∗∗*^*P*  <  0.01.

We also used StarBase 2.0 to predict the potential target genes of miR-155 and found that suppressor of cytokine signaling 1 (SOCS1) might serve as a candidate target of miR-155 with high scores (Figure 4(a)). A dual-luciferase reporter assay indicated that miR-155 mimics could significantly weaken the luciferase activities of the vector carrying SOCS1 (Wt) 3′UTR instead of a mutant-type in Huh7 cell (Figure 4(b)), indicating that miR-155 could bind the 3′UTR of SOCS1 mRNA. Moreover, the mRNA and protein expression of SOCS1 were significantly downregulated in the miR-155stably-overexpressing Huh7 cells compared with control group (Figure 4(c)). Besides, as revealed by the expressing correlation analysis, SOCS1 expression was negative correlated with miR-155 (Figure 4(d)), and positive correlated with CASC2 (Figure 4(e)) in HCC specimens. A rescue assay was conducted to clarify whether CASC2 regulates the biological function of HCCs cell through the miR-155/SOCS1 axis. We transfected CASC2-NC, CASC2-OE, and CASC2-OE + si-SOCS1 into Huh7 cells. The CCK-8 assay demonstrated that SOCS1 knockdown partially inverses the suppression effect of cell proliferation regulated by CASC2. Meanwhile, the transwell assays demonstrated that SOCS1 inhibition led to increased cell migration and invasion of Huh7 cells. Overall, these findings indicated that CASC2 may display the biological activity through regulating the miR-155/SOCS1 axis in HCC.

## 4. Discussion

During recent years, increasing evidence revealed that IncRNAs involve in clinical progression of many tumors including HCC. Several lncRNAs have been reported to play a vital role in regulating the tumorigenesis and cancer progression of HCC. He et al. [16] demonstrated that lncRNA maternally expressed gene 3 (MEG3) was downregulated in HCC and inhibited cell proliferation in vitro and tumor growth in vivo. Yang et al. [17] found that overexpression of HOTAIR could improve the carcinogenic activity of HCC cells and inhibit cell apoptosis. MALAT1 was reported to promote the growth activity and invasiveness of HCC cells. Further research showed MALAT1 served as an oncogene by sponging miR-204 and releasing SIRT1 [18]. Besides, CTBP1-AS2 was associated with the occurrence and progression of HCC. Many studies previously demonstrated that CASC2 could function as a tumor suppressor in human cancers [19]. Jiang et al. [20] revealed that CASC2 could obviously inhibit the glioma cell proliferation and the growth of tumor xenografts in vivo by targeting miR-21. Wang et al. [21] found CASC2 repressed epithelial-mesenchymal transition (EMT) process of HCC cells by regulating the miR-367/FBXW7 axis. There is however limited investigation regarding the development effects and mechanisms by which CASC2 exerts on HCC.

In our study, we found that lncRNA CASC2 was significantly downregulated in HCC tissues compared with adjacent normal tissues and lowly expressed in four HCC cell lines. Then we analyzed the correlation between CASC2 expression and clinicopathological parameters of HCC patients and found that CASC2 were closely associated with TNM stage and lymph nodes metastasis. CASC2 expression was higher at a lower TNM stage compared to a higher TNM stage, and low expression of CASC2 presented a positive lymph node metastasis. Furthermore, Kaplan–Meier assays were performed to explore the prognostic value of CASC2 expression for HCC patients. The analysis indicated that HCC patients with higher expression of CASC2 have a longer OS and DFS than patients with low expression. A cox regression analysis showed that CASC2 expression was an independent prognostic indicator of HCC patients. These results showed that CACS2 might be a potential biomarker for the diagnosis and prognosis prediction of HCC. In order to investigate the biological function of CASC2 in HCC, CASC2 was overexpressed in Huh7 and HepG2 cells by cell transfection. We found that overexpression of CASC2 remarkably inhibited the proliferation, migration, and invasion of Huh7 and HepG2 cells, determined by CCK-8 and Transwell experiments, which indicated the tumor suppressor role of CASC2 in HCC.

MiRNAs are an abundant class of small, noncoding RNAs, which have been identified as important regulators of many biological process. It has been reported that lncRNAs can regulate miRNA expression by acting as competing endogenous RNA (ceRNA) [22]. In recent years, miRNAs were determined to regulate protein-coding gene expression by suppressing mRNA translation or reducing mRNA stability. And many miRNAs are identified to affect cancer phenotype by inhibiting the expression of oncogenes or tumor suppressors [23]. MiR-155 has been implicated in many human cancers, and aberrant expression of miR-155 displays an oncogenic feature. It is reported that miR-155 directly targets and inhibits many genes such as ATG5, SOCS3, SHIP1, and BCL2, which are involved in DNA damage response, cell cycle, hypoxia, inflammation, and tumorigenesis [24–26]. For instance, miR-155 is highly expressed in breast cancer, and high expression levels of miR-155 are associated with tumor subtype, metastasis, and poor survival rate of breast cancer patients [27]. Liu et al. [28] revealed that miR-155 could regulate the expression of PTEN, SOCS6, and SOCS1 protein by targeting the 3′ UTR of their mRNA directly, and inhibition miR-155 decreased cell proliferation and migration in cell lines H1299 and A549. Ahmadvand et al. [29] found that miR-155 is highly expressed in patients with diffuse large B cell lymphoma and directly inhibits HGAL expression.

To further investigate the underlying mechanisms of CASC2, we determined the potential miRNA of CASC2 with StarBase 2.0, and the results showed there was a binding sequence between CASC2 and miR-155. As shown in the luciferase reporter assay, the activity of luciferase in WT-CASC2 was distinctly inhibited by miR-155, which indicated that CASC2 could directly bound to miR-155. Then, we observed the distinct increase in miR-155 expression in 4 HCC cell lines, and found that overexpression of CASC2 could inhibit the level of miR-155 in Huh7 cells markedly. Also, we found that miR-155 was upregulated in HCC tissues, and correlation analysis presented a negative correlation between CASC2 and miR-155 expression in HCC tissues. Taken together, CASC2 may exhibit its tumor suppressor roles by acting as a competing endogenous RNA to sponge miR-155.

Growing studies have reported that SOCS1 displayed a displayed a dysregulated expression in gastric cancer, HCC, breast cancer and pancreatic cancer [30–32]. In this study, we found that SOCS1 was predicted as a candidate target of miR-155, and dual-luciferase reporter assays confirmed the direct targeting by miR-155 over SOCS1. Furthermore, SOCS1 was low-expressed in HCC tissue samples and cell lines and exhibited a negative relationship with miR-155 and a positive relation with CASC2 in HCC samples. The mRNA and protein levels of SOCS1 were significantly upregulated compared with the control group after miR-155 was overexpressed in Huh7. Finally, we performed rescue experiments, finding that SOCS1 inhibition partially inverses the suppression effect of cell proliferation regulated by CASC2. And transwell assays showed that SOCS1 knockdown led to increased cell migration and invasion of Huh7 cells. Functionally, these results confirmed that CASC2 could function as a tumor suppressor by acting as a ceRNA to bind to miR-155 and downregulate the expression of SOCS1.

In conclusion, our findings uncovered that CASC2 could act as a tumor suppressor gene and inhibit cell proliferation, migration, and invasion through binding to miR-155, thus increasing the expression of its target gene SOCS1, demonstrating the ceRNA function of CASC2. In our study, we have partially elucidated the role of the CASC2/miR-155/SOCS1 axis in HCC development.

## Figures and Tables

**Table 1:** Univariate and multivariate analyses for correlation of CASC2 expression with OS of HCC patients.

Variable	Univariate Cox's regression analysis		Multivariate Cox's regression analysis	
	Hazard ratio (95% CI)	*P* value	Hazard ratio (95% CI)	*P* value
CASC2 expression (high vs. low)	1.237 (0.775–2.044)	0.008	1.879 (0.711–2.425)	0.004^*∗*^
Sex (male vs. female)	2.432 (1.337–3.212)	0.304	—	—
Age (<60 years vs. ≥60 years)	2.099 (1.225–3.108)	0.112	—	—
Tumor size (≥5 cm vs. <5 cm)	0.974 (0.582–1.706)	0.284	—	—
TNM staging (I∼II vs. III∼IV)	1.262 (0.897–1.981)	0.016	1.765 (0.892–2.241)	0.012^*∗*^
Differentiation (poor vs. good/moderate)	1.417 (0.692–2.288)	0.492	—	—
Lymph node metastasis (yes vs. No)	1.696 (1.125–2.198)	0.005	1.233 (1.108–2.491)	0.002^*∗*^

*P* value was acquired by Cox proportional hazards regression. ^*∗*^Statistically significant (*P* < 0.05).

## Data Availability

The datasets used and analyzed during the current study are available from the corresponding author on reasonable request.
